# Supplementary material for: Resolving the full spectrum of human genome variation using Linked-Reads
Source: Genome Res. 2019 Apr;29(4):635–45. doi: 10.1101/gr.234443.118 (PMC6442396; doi:10.1101/gr.234443.118)
Supplement: Supplemental Material [file supp_gr.234443.118_Supplemental_longranger-2.2.zip › longranger-2.2.tar.bz2!/lr2.2-public/lib/stan/cmdstan/stan/doc/api/html/categorical__argument_8hpp_source.html]

Stan: src/stan/gm/arguments/categorical\_argument.hpp Source File


|  |  |
| --- | --- |
|  | Stan  2.5.0  probability, sampling & optimization |


- Main Page
- Related Pages
- Namespaces
- Classes
- Files

- File List
- File Members

All Classes Namespaces Files Functions Variables Typedefs Enumerations Enumerator Friends Macros Pages

- src
- stan
- gm
- arguments

categorical\_argument.hpp

Go to the documentation of this file.

1 #ifndef STAN\_\_GM\_\_ARGUMENTS\_\_CATEGORY\_\_ARGUMENT\_\_BETA

2 #define STAN\_\_GM\_\_ARGUMENTS\_\_CATEGORY\_\_ARGUMENT\_\_BETA

3

4 #include <vector>

5 #include <stan/gm/arguments/argument.hpp>

6

7 namespace stan {

8

9  namespace gm {

10

11  class categorical\_argument: public argument {

12

13  public:

14

15  virtual ~categorical\_argument() {

16  for (std::vector<argument\*>::iterator it = \_subarguments.begin();

17  it != \_subarguments.end(); ++it) {

18  delete \*it;

19  }

20

21  \_subarguments.clear();

22  }

23

24  void print(std::ostream\* s, const int depth, const std::string prefix) {

25  if (!s)

26  return;

27  std::string indent(compute\_indent(depth), ' ');

28  \*s << prefix << indent << \_name << std::endl;

29

30  for (std::vector<argument\*>::iterator it = \_subarguments.begin();

31  it != \_subarguments.end(); ++it)

32  (\*it)->print(s, depth + 1, prefix);

33  }

34

35  void print\_help(std::ostream\* s, const int depth, const bool recurse) {

36

37  if (!s)

38  return;

39

40  std::string indent(indent\_width \* depth, ' ');

41  std::string subindent(indent\_width, ' ');

42

43  \*s << indent << \_name << std::endl;

44  \*s << indent << subindent << \_description << std::endl;

45  if (\_subarguments.size() > 0) {

46  \*s << indent << subindent << "Valid subarguments:";

47

48  std::vector<argument\*>::iterator it = \_subarguments.begin();

49  \*s << " " << (\*it)->name();

50  ++it;

51

52  for (; it != \_subarguments.end(); ++it)

53  \*s << ", " << (\*it)->name();

54  \*s << std::endl << std::endl;

55

56  if (recurse) {

57  for (std::vector<argument\*>::iterator it = \_subarguments.begin();

58  it != \_subarguments.end(); ++it)

59  (\*it)->print\_help(s, depth + 1, true);

60  }

61  }

62  else {

63  \*s << std::endl;

64  }

65

66  }

67

68  bool parse\_args(std::vector<std::string>& args, std::ostream\* out,

69  std::ostream\* err, bool& help\_flag) {

70

71  bool good\_arg = true;

72  bool valid\_arg = true;

73

74  while(good\_arg) {

75  if (args.size() == 0)

76  return valid\_arg;

77

78  good\_arg = false;

79

80  std::string cat\_name = args.back();

81

82  if (cat\_name == "help") {

83  print\_help(out, 0, false);

84  help\_flag |= true;

85  args.clear();

86  return true;

87  } else if (cat\_name == "help-all") {

88  print\_help(out, 0, true);

89  help\_flag |= true;

90  args.clear();

91  return true;

92  }

93

94  std::string val\_name;

95  std::string val;

96  split\_arg(cat\_name, val\_name, val);

97

98  if (\_subarguments.size() == 0)

99  valid\_arg = true;

100  for (std::vector<argument\*>::iterator it = \_subarguments.begin();

101  it != \_subarguments.end(); ++it) {

102  if ( (\*it)->name() == cat\_name) {

103  args.pop\_back();

104  valid\_arg &= (\*it)->parse\_args(args, out, err, help\_flag);

105  good\_arg = true;

106  break;

107  } else if ( (\*it)->name() == val\_name ) {

108  valid\_arg &= (\*it)->parse\_args(args, out, err, help\_flag);

109  good\_arg = true;

110  break;

111  } else {

112  good\_arg = false;

113  }

114  }

115  }

116  return valid\_arg;

117  };

118

119  virtual void probe\_args(argument\* base\_arg, std::stringstream& s) {

120  for (std::vector<argument\*>::iterator it = \_subarguments.begin();

121  it != \_subarguments.end(); ++it) {

122  (\*it)->probe\_args(base\_arg, s);

123  }

124  }

125

126  void find\_arg(std::string name,

127  std::string prefix,

128  std::vector<std::string>& valid\_paths) {

129

130  argument::find\_arg(name, prefix, valid\_paths);

131

132  prefix += \_name + " ";

133  for (std::vector<argument\*>::iterator it = \_subarguments.begin();

134  it != \_subarguments.end(); ++it)

135  (\*it)->find\_arg(name, prefix, valid\_paths);

136

137  }

138

139  std::vector<argument\*>& subarguments() {

140  return \_subarguments;

141  }

142

143  argument\* arg(const std::string name) {

144  for (std::vector<argument\*>::iterator it = \_subarguments.begin();

145  it != \_subarguments.end(); ++it)

146  if ( name == (\*it)->name() )

147  return (\*it);

148  return 0;

149  }

150

151  protected:

152

153  std::vector<argument\*> \_subarguments;

154

155  };

156

157  } // gm

158

159 } // stan

160

161 #endif

stan::gm::categorical\_argument::~categorical\_argument

virtual ~categorical\_argument()

**Definition:** categorical\_argument.hpp:15

stan::gm::categorical\_argument::parse\_args

bool parse\_args(std::vector< std::string > &args, std::ostream \*out, std::ostream \*err, bool &help\_flag)

**Definition:** categorical\_argument.hpp:68

stan::gm::categorical\_argument::arg

argument \* arg(const std::string name)

**Definition:** categorical\_argument.hpp:143

stan::gm::categorical\_argument::probe\_args

virtual void probe\_args(argument \*base\_arg, std::stringstream &s)

**Definition:** categorical\_argument.hpp:119

argument.hpp

stan::gm::categorical\_argument

**Definition:** categorical\_argument.hpp:11

stan::gm::categorical\_argument::\_subarguments

std::vector< argument \* > \_subarguments

**Definition:** categorical\_argument.hpp:153

stan::gm::argument::find\_arg

virtual void find\_arg(std::string name, std::string prefix, std::vector< std::string > &valid\_paths)

**Definition:** argument.hpp:46

stan::gm::categorical\_argument::subarguments

std::vector< argument \* > & subarguments()

**Definition:** categorical\_argument.hpp:139

stan::gm::argument::split\_arg

static void split\_arg(const std::string &arg, std::string &name, std::string &value)

**Definition:** argument.hpp:54

stan::gm::argument::compute\_indent

int compute\_indent(const int depth)

**Definition:** argument.hpp:71

stan::gm::categorical\_argument::print\_help

void print\_help(std::ostream \*s, const int depth, const bool recurse)

**Definition:** categorical\_argument.hpp:35

stan::gm::categorical\_argument::print

void print(std::ostream \*s, const int depth, const std::string prefix)

**Definition:** categorical\_argument.hpp:24

stan::gm::categorical\_argument::find\_arg

void find\_arg(std::string name, std::string prefix, std::vector< std::string > &valid\_paths)

**Definition:** categorical\_argument.hpp:126

stan::gm::argument::\_name

std::string \_name

**Definition:** argument.hpp:76

stan::gm::argument::indent\_width

int indent\_width

**Definition:** argument.hpp:79

stan::gm::argument::name

std::string name() const

**Definition:** argument.hpp:26

stan::gm::argument

**Definition:** argument.hpp:12

stan::gm::argument::\_description

std::string \_description

**Definition:** argument.hpp:77


---

[ Stan Home Page ]

*© 2011–2014,
Stan Development Team.*
